# 2-Chloro-8-methyl-3-[(pyrimidin-4-yl­oxy)meth­yl]quinoline

**DOI:** 10.1107/S1600536810011694

**Published:** 2010-04-02

**Authors:** F. Nawaz Khan, S. Mohana Roopan, Venkatesha R. Hathwar, Mehmet Akkurt

**Affiliations:** a Organic and Medicinal Chemistry Research Laboratory, Organic Chemistry Division, School of Advanced Sciences, VIT University, Vellore 632 014, Tamil Nadu, India; b Solid State and Structural Chemistry Unit, Indian Institute of Science, Bangalore 560 012, Karnataka, India; c Department of Physics, Faculty of Arts and Sciences, Erciyes University, 38039 Kayseri, Turkey

## Abstract

In the title compound, C_15_H_12_ClN_3_O, the quinoline ring system is essentially planar, with a maximum deviation of 0.017 (1) Å. The crystal packing is stabilized by π–π stacking inter­actions between the quinoline rings of adjacent mol­ecule, with a centroid–centroid distance of 3.5913 (8) Å. A weak C—H⋯π contact is also observed between mol­ecules.

## Related literature

For pyrimidine analogues, see: Svenstrup *et al.* (2008 ▶). For quinoline analogues, see: Roopan & Khan (2009 ▶); Khan *et al.* (2009 ▶, 2010*a* ▶,*b* ▶). For the biological activity and mode of action of alkyl­ating agent, see: Singer (1986 ▶). For the synthesis and regioselective alkyl­ation of 4(3*H*)-pyrimidone, see: Roopan *et al.* (2010 ▶). For bond-length data, see: Allen *et al.* (1987 ▶). For a structural discussion on hydrogen bonding, see: Bernstein *et al.* (1995 ▶).
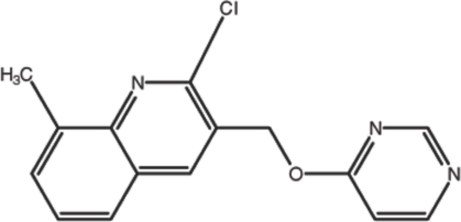


## Experimental

### 

#### Crystal data

C_15_H_12_ClN_3_O
*M* *_r_* = 285.73
Monoclinic, 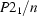

*a* = 11.9975 (2) Å
*b* = 8.45037 (15) Å
*c* = 12.95869 (19) Å
β = 96.7619 (16)°
*V* = 1304.66 (4) Å^3^
*Z* = 4
Mo *K*α radiation
μ = 0.29 mm^−1^
*T* = 295 K
0.23 × 0.18 × 0.15 mm

#### Data collection

Oxford Xcalibur Eos (Nova) CCD detector diffractometer
Absorption correction: multi-scan (*CrysAlis PRO RED*; Oxford Diffraction, 2009 ▶) *T*_min_ = 0.936, *T*_max_ = 0.958
13753 measured reflections
2564 independent reflections
2005 reflections with *I* > 2σ(*I*)
*R*_int_ = 0.031

#### Refinement

*R*[*F* ^2^ > 2σ(*F* ^2^)] = 0.033
*wR*(*F* ^2^) = 0.089
*S* = 1.07
2564 reflections
182 parameters
H-atom parameters constrained
Δρ_max_ = 0.18 e Å^−3^
Δρ_min_ = −0.20 e Å^−3^

### 

Data collection: *CrysAlis PRO CCD* (Oxford Diffraction, 2009 ▶); cell refinement: *CrysAlis PRO CCD*; data reduction: *CrysAlis PRO RED* (Oxford Diffraction, 2009 ▶); program(s) used to solve structure: *SHELXS97* (Sheldrick, 2008 ▶); program(s) used to refine structure: *SHELXL97* (Sheldrick, 2008 ▶); molecular graphics: *ORTEP-3 for Windows* (Farrugia, 1997 ▶); software used to prepare material for publication: *WinGX* (Farrugia, 1999 ▶) and *PLATON* (Spek, 2009 ▶).

## Supplementary Material

Crystal structure: contains datablocks global, I. DOI: 10.1107/S1600536810011694/fj2289sup1.cif

Structure factors: contains datablocks I. DOI: 10.1107/S1600536810011694/fj2289Isup2.hkl

Additional supplementary materials:  crystallographic information; 3D view; checkCIF report

## Figures and Tables

**Table 1:** Hydrogen-bond geometry (Å, °) *Cg*3 is the centroid of the C4–C9 ring.

*D*—H⋯*A*	*D*—H	H⋯*A*	*D*⋯*A*	*D*—H⋯*A*
C10—H10*A*⋯*Cg*3^i^	0.97	2.72	3.5132 (15)	140

Symmetry code: (i) 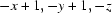.

**Fig. 1.**
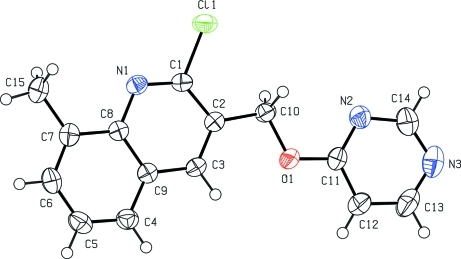
The molecular structure of the title molecule with the atom-numbering scheme. Displacement ellipsoids are drawn at the 50% probability level.

**Fig. 2.**
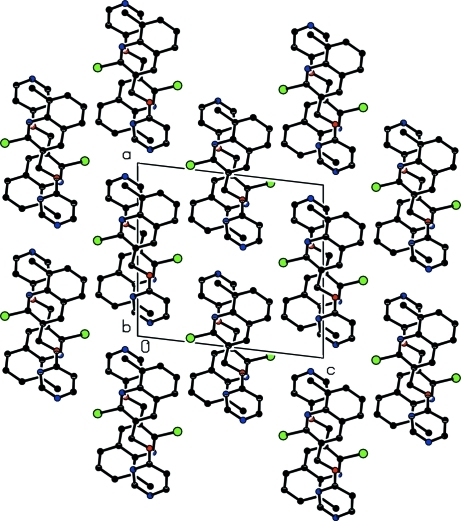
Packing diagram of the title compound viewed down b-axis. All H atoms have been omitted for clarity.

## supplementary crystallographic information

### Comment

Alkylating agents have been studied extensively both for their biological effects and for their mode of action (Singer *et al.*, 1986). There have been over the past 25 years a veritable deluge of reviews, often focusing on a single aspect or agent or adduct. Direct alkylation of oxygens in pyrimidine nucleosides, under physiological conditions, has been known only since the middle 1970s. The pyrimidine analogues (Svenstrup *et al.*, 2008) such as naturally occurring azacamptothecin based molecule have been focused of great interest by reason of their diversified biological activities. Thus, modifications of biologically active azacamptothecin synthons may lead to achieve the highly expected effective drugs. In connection with the program of synthesis and regioselective alkylation of 4(3*H*)-pyrimidone (Roopan *et al.*, 2010), we report herein the synthesis of 2-chloro-8-methyl-3-[(pyrimidin-4-yloxy)methyl]quinoline.

In the molecule of the title compound, Fig. 1, bond lengths and angles are in normal ranges (Allen *et al.*, 1987). The quinoline ring system (N1/C1–C9) is essentially planar, with a maximum deviation of -0.017 (1) Å for atom C1. The quinoline system (N1/C1–C9) makes a dihedral angle of 4.99 (6)° with the piyrimidone ring (N2/N3/C11–C14).

In the title molecule, there is a weak intramolecular C—H···O interaction, generating an *S*(5) graph-set motif (Bernstein *et al.*, 1995) (Table 1). The crystal packing is stabilized by π-π stacking interactions between the benzene rings of the quinoline ring system of the molecules related by the symmetry operator (1-x, 1-y, -z) [centroid-to-centroid distance = 3.5913 (8) Å]. In addition, a weak C—H···π contact is also observed between molecules (Table 1). The packing diagram viewing down the b-axis is shown in Fig. 2.

### Experimental

To a mixed well solution of 4(3*H*)-pyrimidone (96 mg, 1 mmol, in 5 ml DMSO), NaH (25 mg, 1 mmol) and 2-chloro-3-(chloromethyl)-8-methylquinoline (225 mg, 1 mmol) were added and the resulting mixture was refluxed for 1 h. Completion of the reaction was monitored by TLC. After the completion of the reaction, cooled and removed the excess of solvent under reduced pressure. Crushed ice was mixed with the residue. White solid was formed, filtered and dried, purified by column chromatography using hexane and ethylacetate as the eluant. The low polar compound was subjected into crystallization by solvent evaporation from a solution of the compound in chloroform.

### Refinement

All H atoms were placed in geometrically idealised positions and constrained to ride on their parent atoms (C—H = 0.93, 0.96 and 0.97 Å) and U_iso_(H) values were taken to be equal to 1.2 U_eq_(C) for aromatic and methylene H atoms and 1.5U_eq_(C) for methyl H atoms.

### Crystal data

**Table:**

C_15_H_12_ClN_3_O	*F*(000) = 592
*M**_r_* = 285.73	*D*_x_ = 1.455 Mg m^−^^3^
Monoclinic, *P*2_1_/*n*	Mo *K*α radiation, λ = 0.71073 Å
Hall symbol: -P 2yn	Cell parameters from 1065 reflections
*a* = 11.9975 (2) Å	θ = 2.5–26.0°
*b* = 8.45037 (15) Å	µ = 0.29 mm^−^^1^
*c* = 12.95869 (19) Å	*T* = 295 K
β = 96.7619 (16)°	Prism, colourless
*V* = 1304.66 (4) Å^3^	0.23 × 0.18 × 0.15 mm
*Z* = 4	

### Data collection

**Table:**

Oxford Xcalibur Eos (Nova) CCD detector diffractometer	2564 independent reflections
Radiation source: Enhance (Mo) X-ray Source	2005 reflections with *I* > 2σ(*I*)
graphite	*R*_int_ = 0.031
ω scans	θ_max_ = 26.0°, θ_min_ = 2.5°
Absorption correction: multi-scan (*CrysAlis PRO* RED; Oxford Diffraction, 2009)	*h* = −14→14
*T*_min_ = 0.936, *T*_max_ = 0.958	*k* = −10→10
13753 measured reflections	*l* = −15→15

### Refinement

**Table:**

Refinement on *F*^2^	Primary atom site location: structure-invariant direct methods
Least-squares matrix: full	Secondary atom site location: difference Fourier map
*R*[*F*^2^ > 2σ(*F*^2^)] = 0.033	Hydrogen site location: inferred from neighbouring sites
*wR*(*F*^2^) = 0.089	H-atom parameters constrained
*S* = 1.07	*w* = 1/[σ^2^(*F*_o_^2^) + (0.0503*P*)^2^] where *P* = (*F*_o_^2^ + 2*F*_c_^2^)/3
2564 reflections	(Δ/σ)_max_ < 0.001
182 parameters	Δρ_max_ = 0.18 e Å^−^^3^
0 restraints	Δρ_min_ = −0.20 e Å^−^^3^

### Special details

**Table:**

Geometry. Bond distances, angles etc. have been calculated using the rounded fractional coordinates. All su's are estimated from the variances of the (full) variance-covariance matrix. The cell esds are taken into account in the estimation of distances, angles and torsion angles
Refinement. Refinement on *F*^2^ for ALL reflections except those flagged by the user for potential systematic errors. Weighted *R*-factors wR and all goodnesses of fit *S* are based on *F*^2^, conventional *R*-factors *R* are based on *F*, with *F* set to zero for negative *F*^2^. The observed criterion of *F*^2^ > σ(*F*^2^) is used only for calculating -*R*-factor-obs etc. and is not relevant to the choice of reflections for refinement. *R*-factors based on *F*^2^ are statistically about twice as large as those based on *F*, and *R*-factors based on ALL data will be even larger.

### Fractional atomic coordinates and isotropic or equivalent isotropic displacement parameters (Å^2^)

**Table:**

	*x*	*y*	*z*	*U*_iso_*/*U*_eq_	
Cl1	0.52448 (3)	0.22861 (5)	−0.21533 (3)	0.0452 (2)	
O1	0.35909 (9)	0.13571 (11)	0.06428 (7)	0.0413 (4)	
N1	0.66190 (10)	0.38546 (12)	−0.08362 (8)	0.0315 (4)	
N2	0.22497 (11)	−0.02532 (14)	−0.02386 (9)	0.0398 (4)	
N3	0.08616 (12)	−0.14701 (16)	0.06771 (13)	0.0569 (6)	
C1	0.57162 (12)	0.30022 (16)	−0.09125 (10)	0.0297 (4)	
C2	0.50756 (12)	0.25855 (14)	−0.01007 (10)	0.0282 (4)	
C3	0.54805 (12)	0.31329 (15)	0.08638 (10)	0.0299 (4)	
C4	0.69272 (13)	0.46034 (15)	0.20041 (11)	0.0365 (5)	
C5	0.78847 (14)	0.54796 (16)	0.20983 (12)	0.0418 (5)	
C6	0.84248 (13)	0.58407 (16)	0.12263 (12)	0.0399 (5)	
C7	0.80215 (13)	0.53220 (16)	0.02491 (11)	0.0345 (5)	
C8	0.70266 (12)	0.43947 (15)	0.01381 (10)	0.0291 (4)	
C9	0.64712 (12)	0.40438 (15)	0.10159 (10)	0.0296 (4)	
C10	0.40366 (12)	0.16051 (16)	−0.03229 (10)	0.0327 (5)	
C11	0.26755 (13)	0.04265 (16)	0.06302 (11)	0.0344 (5)	
C12	0.22415 (15)	0.02264 (18)	0.15667 (12)	0.0459 (6)	
C13	0.13310 (16)	−0.0742 (2)	0.15376 (15)	0.0551 (7)	
C14	0.13543 (15)	−0.11672 (19)	−0.01572 (14)	0.0514 (6)	
C15	0.86040 (14)	0.57135 (18)	−0.06845 (13)	0.0465 (6)	
H3	0.50970	0.29010	0.14280	0.0360*	
H4	0.65740	0.43730	0.25880	0.0440*	
H5	0.81850	0.58440	0.27500	0.0500*	
H6	0.90750	0.64490	0.13120	0.0480*	
H10A	0.34910	0.21490	−0.08100	0.0390*	
H10B	0.42140	0.05980	−0.06240	0.0390*	
H12	0.25520	0.07210	0.21750	0.0550*	
H13	0.10170	−0.09080	0.21520	0.0660*	
H14	0.10390	−0.16500	−0.07680	0.0620*	
H15A	0.92390	0.63810	−0.04780	0.0700*	
H15B	0.80920	0.62550	−0.11900	0.0700*	
H15C	0.88530	0.47550	−0.09820	0.0700*	

### Atomic displacement parameters (Å^2^)

**Table:**

	*U*^11^	*U*^22^	*U*^33^	*U*^12^	*U*^13^	*U*^23^
Cl1	0.0438 (3)	0.0642 (3)	0.0278 (2)	−0.0083 (2)	0.0045 (2)	−0.0068 (2)
O1	0.0385 (7)	0.0543 (6)	0.0322 (6)	−0.0162 (5)	0.0092 (5)	−0.0009 (5)
N1	0.0286 (7)	0.0364 (6)	0.0301 (6)	0.0026 (5)	0.0059 (5)	0.0019 (5)
N2	0.0355 (8)	0.0428 (7)	0.0406 (7)	−0.0044 (6)	0.0027 (6)	0.0005 (6)
N3	0.0425 (10)	0.0507 (9)	0.0792 (11)	−0.0086 (7)	0.0143 (8)	0.0065 (8)
C1	0.0293 (8)	0.0340 (7)	0.0259 (7)	0.0036 (6)	0.0034 (6)	−0.0007 (5)
C2	0.0268 (8)	0.0278 (7)	0.0303 (7)	0.0045 (6)	0.0046 (6)	0.0023 (6)
C3	0.0307 (9)	0.0319 (7)	0.0280 (7)	0.0033 (6)	0.0078 (6)	0.0029 (6)
C4	0.0409 (10)	0.0368 (8)	0.0313 (8)	0.0009 (7)	0.0025 (7)	0.0009 (6)
C5	0.0469 (11)	0.0396 (8)	0.0362 (8)	0.0005 (7)	−0.0062 (7)	−0.0046 (6)
C6	0.0315 (9)	0.0359 (8)	0.0507 (9)	−0.0041 (7)	−0.0015 (7)	−0.0012 (7)
C7	0.0306 (9)	0.0316 (8)	0.0418 (8)	0.0017 (6)	0.0060 (7)	0.0014 (6)
C8	0.0279 (8)	0.0285 (7)	0.0309 (7)	0.0037 (6)	0.0035 (6)	0.0006 (5)
C9	0.0297 (8)	0.0279 (7)	0.0310 (7)	0.0037 (6)	0.0029 (6)	0.0021 (5)
C10	0.0310 (9)	0.0386 (8)	0.0288 (7)	−0.0009 (6)	0.0054 (6)	0.0011 (6)
C11	0.0295 (9)	0.0343 (8)	0.0399 (8)	−0.0010 (6)	0.0063 (7)	0.0045 (6)
C12	0.0498 (11)	0.0489 (9)	0.0417 (9)	−0.0058 (8)	0.0165 (8)	0.0004 (7)
C13	0.0533 (12)	0.0523 (10)	0.0647 (12)	−0.0042 (9)	0.0281 (10)	0.0087 (9)
C14	0.0422 (11)	0.0501 (10)	0.0605 (11)	−0.0089 (8)	0.0008 (9)	−0.0019 (8)
C15	0.0387 (10)	0.0498 (10)	0.0526 (10)	−0.0108 (8)	0.0119 (8)	0.0019 (7)

### Geometric parameters (Å, °)

**Table:**

Cl1—C1	1.7485 (14)	C7—C8	1.421 (2)
O1—C10	1.4330 (16)	C7—C15	1.504 (2)
O1—C11	1.3493 (18)	C8—C9	1.4158 (19)
N1—C1	1.2948 (18)	C11—C12	1.386 (2)
N1—C8	1.3770 (17)	C12—C13	1.362 (3)
N2—C11	1.3126 (18)	C3—H3	0.9300
N2—C14	1.337 (2)	C4—H4	0.9300
N3—C13	1.338 (2)	C5—H5	0.9300
N3—C14	1.317 (2)	C6—H6	0.9300
C1—C2	1.4184 (19)	C10—H10A	0.9700
C2—C3	1.3672 (18)	C10—H10B	0.9700
C2—C10	1.496 (2)	C12—H12	0.9300
C3—C9	1.410 (2)	C13—H13	0.9300
C4—C5	1.360 (2)	C14—H14	0.9300
C4—C9	1.4134 (19)	C15—H15A	0.9600
C5—C6	1.401 (2)	C15—H15B	0.9600
C6—C7	1.373 (2)	C15—H15C	0.9600
			
Cl1···C15^i^	3.5264 (17)	C5···H10A^vi^	2.9800
Cl1···H10A	2.8900	C6···H10A^vi^	2.8600
Cl1···H10B	2.8400	C7···H10A^vi^	2.9500
Cl1···H14^ii^	3.0800	C10···H10B^v^	2.9600
O1···H3	2.3600	C11···H15B^vi^	3.0700
N1···H15B	2.7600	C12···H15B^vi^	3.0300
N1···H15C	2.8100	H3···O1	2.3600
N2···H10A	2.6700	H3···H4	2.5100
N2···H10B	2.5700	H4···H3	2.5100
N2···H4^iii^	2.9300	H4···N2^ix^	2.9300
N3···H15A^iv^	2.9400	H5···C3^x^	2.9800
N3···H15C^v^	2.8200	H6···H15A	2.3500
C1···C3^vi^	3.5712 (19)	H10A···Cl1	2.8900
C1···C11^v^	3.476 (2)	H10A···N2	2.6700
C2···C8^vi^	3.5842 (19)	H10A···C5^vi^	2.9800
C2···C9^vi^	3.5278 (18)	H10A···C6^vi^	2.8600
C3···C1^vi^	3.5712 (19)	H10A···C7^vi^	2.9500
C7···C14^v^	3.595 (2)	H10B···Cl1	2.8400
C7···C10^vi^	3.592 (2)	H10B···N2	2.5700
C8···C2^vi^	3.5842 (19)	H10B···C2^v^	2.9400
C8···C14^v^	3.347 (2)	H10B···C10^v^	2.9600
C9···C2^vi^	3.5278 (18)	H10B···H10B^v^	2.5400
C10···C7^vi^	3.592 (2)	H14···Cl1^xi^	3.0800
C10···C10^v^	3.598 (2)	H15A···N3^xii^	2.9400
C11···C1^v^	3.476 (2)	H15A···H6	2.3500
C14···C8^v^	3.347 (2)	H15B···N1	2.7600
C14···C7^v^	3.595 (2)	H15B···C11^vi^	3.0700
C15···Cl1^vii^	3.5264 (17)	H15B···C12^vi^	3.0300
C2···H10B^v^	2.9400	H15C···N1	2.8100
C3···H5^viii^	2.9800	H15C···N3^v^	2.8200
			
C10—O1—C11	117.49 (10)	N3—C13—C12	123.83 (17)
C1—N1—C8	117.26 (11)	N2—C14—N3	128.27 (16)
C11—N2—C14	114.85 (13)	C2—C3—H3	119.00
C13—N3—C14	114.15 (15)	C9—C3—H3	119.00
Cl1—C1—N1	116.09 (10)	C5—C4—H4	120.00
Cl1—C1—C2	116.83 (10)	C9—C4—H4	120.00
N1—C1—C2	127.08 (12)	C4—C5—H5	120.00
C1—C2—C3	115.39 (12)	C6—C5—H5	120.00
C1—C2—C10	120.44 (11)	C5—C6—H6	119.00
C3—C2—C10	124.17 (12)	C7—C6—H6	119.00
C2—C3—C9	121.02 (12)	O1—C10—H10A	110.00
C5—C4—C9	119.75 (14)	O1—C10—H10B	110.00
C4—C5—C6	120.81 (14)	C2—C10—H10A	110.00
C5—C6—C7	121.88 (14)	C2—C10—H10B	110.00
C6—C7—C8	118.03 (13)	H10A—C10—H10B	108.00
C6—C7—C15	121.68 (14)	C11—C12—H12	122.00
C8—C7—C15	120.29 (13)	C13—C12—H12	122.00
N1—C8—C7	118.62 (12)	N3—C13—H13	118.00
N1—C8—C9	121.15 (12)	C12—C13—H13	118.00
C7—C8—C9	120.23 (12)	N2—C14—H14	116.00
C3—C9—C4	122.63 (12)	N3—C14—H14	116.00
C3—C9—C8	118.08 (12)	C7—C15—H15A	109.00
C4—C9—C8	119.29 (13)	C7—C15—H15B	109.00
O1—C10—C2	107.52 (10)	C7—C15—H15C	109.00
O1—C11—N2	119.96 (13)	H15A—C15—H15B	109.00
O1—C11—C12	116.72 (13)	H15A—C15—H15C	109.00
N2—C11—C12	123.31 (14)	H15B—C15—H15C	109.00
C11—C12—C13	115.58 (15)		
			
C11—O1—C10—C2	−176.75 (11)	C2—C3—C9—C4	−178.71 (13)
C10—O1—C11—N2	2.21 (19)	C2—C3—C9—C8	0.9 (2)
C10—O1—C11—C12	−178.68 (13)	C9—C4—C5—C6	0.2 (2)
C8—N1—C1—Cl1	−178.15 (10)	C5—C4—C9—C3	−179.87 (13)
C8—N1—C1—C2	1.6 (2)	C5—C4—C9—C8	0.6 (2)
C1—N1—C8—C7	179.47 (12)	C4—C5—C6—C7	−0.5 (2)
C1—N1—C8—C9	−0.69 (19)	C5—C6—C7—C8	0.0 (2)
C14—N2—C11—O1	179.03 (13)	C5—C6—C7—C15	179.97 (14)
C14—N2—C11—C12	0.0 (2)	C6—C7—C8—N1	−179.40 (12)
C11—N2—C14—N3	−0.5 (2)	C6—C7—C8—C9	0.8 (2)
C14—N3—C13—C12	−0.3 (2)	C15—C7—C8—N1	0.6 (2)
C13—N3—C14—N2	0.6 (3)	C15—C7—C8—C9	−179.20 (13)
Cl1—C1—C2—C3	178.55 (10)	N1—C8—C9—C3	−0.48 (19)
Cl1—C1—C2—C10	−1.18 (17)	N1—C8—C9—C4	179.12 (12)
N1—C1—C2—C3	−1.2 (2)	C7—C8—C9—C3	179.36 (12)
N1—C1—C2—C10	179.08 (13)	C7—C8—C9—C4	−1.0 (2)
C1—C2—C3—C9	−0.14 (19)	O1—C11—C12—C13	−178.79 (14)
C10—C2—C3—C9	179.58 (12)	N2—C11—C12—C13	0.3 (2)
C1—C2—C10—O1	178.90 (11)	C11—C12—C13—N3	−0.1 (3)
C3—C2—C10—O1	−0.80 (18)		

Symmetry codes: (i) −*x*+3/2, *y*−1/2, −*z*−1/2; (ii) −*x*+1/2, *y*+1/2, −*z*−1/2; (iii) *x*−1/2, −*y*+1/2, *z*−1/2; (iv) *x*−1, *y*−1, *z*; (v) −*x*+1, −*y*, −*z*; (vi) −*x*+1, −*y*+1, −*z*; (vii) −*x*+3/2, *y*+1/2, −*z*−1/2; (viii) −*x*+3/2, *y*−1/2, −*z*+1/2; (ix) *x*+1/2, −*y*+1/2, *z*+1/2; (x) −*x*+3/2, *y*+1/2, −*z*+1/2; (xi) −*x*+1/2, *y*−1/2, −*z*−1/2; (xii) *x*+1, *y*+1, *z*.

### Hydrogen-bond geometry (Å, °)

**Table:**

Cg3 is the centroid of the C4–C9 ring.

**Table:**

*D*—H···*A*	*D*—H	H···*A*	*D*···*A*	*D*—H···*A*
C3—H3···O1	0.93	2.36	2.7056 (17)	102
C10—H10A···Cg3^vi^	0.97	2.72	3.5132 (15)	140

Symmetry codes: (vi) −*x*+1, −*y*+1, −*z*.
